# An fMRI Study of Neuronal Specificity in Acupuncture: The Multiacupoint Siguan and Its Sham Point

**DOI:** 10.1155/2014/103491

**Published:** 2014-11-26

**Authors:** Yi Shan, Zhi-qun Wang, Zhi-lian Zhao, Mo Zhang, Shi-lei Hao, Jian-yang Xu, Bao-ci Shan, Jie Lu, Kun-cheng Li

**Affiliations:** ^1^Department of Radiology, Xuanwu Hospital of Capital Medical University, 45 Changchunjie, Xicheng District, Beijing 100053, China; ^2^Beijing Key Laboratory of Magnetic Resonance Imaging and Brain Informatics, Beijing 100053, China; ^3^General Hospital of Chinese People's Armed Police Forces, Beijing 100053, China; ^4^Institute of High Energy Physics, Chinese Academy of Sciences, Beijing 100053, China

## Abstract

Clarifying the intrinsic mechanisms of acupuncture's clinical effects has recently been gaining popularity. Here, we choose the Siguan acupoint (a combination of bilateral LI4 and Liv3) and its sham point to evaluate multiacupoint specificity. Thirty-one healthy volunteers were randomly divided into real acupoint (21 subjects) and sham acupoint (10 subjects) groups. Our study used a single block experimental design to avoid the influence of posteffects. Functional magnetic resonance imaging data were acquired during acupuncture stimulation. Results showed extensive increase in neuronal activities with Siguan acupuncture and significant differences between stimulation at real and sham points. Brain regions that were activated more by real acupuncture stimulation than by sham point acupuncture included somatosensory cortex (the superior parietal lobule and postcentral gyrus), limbic-paralimbic system (the calcarine gyrus, precuneus, cingulate cortex, and parahippocampal gyrus), visual-related cortex (the fusiform and occipital gyri), basal ganglia, and the cerebellum. In this way, our study suggests Siguan may elicit specific activities in human brain.

## 1. Introduction

Acupuncture is a traditional Chinese medicine (TCM) that has been used for thousands of years with empirical evidence of effectiveness. Recently, clarifying the intrinsic mechanisms of its clinical effects has become increasingly popular research. According to the traditional theory of acupuncture, stimulation at specific acupoints will produce effective bodily responses that can be used to treat certain diseases.

Much research has been devoted to demonstrating the mechanisms underlying acupoint specificity. Since the 1990s, development in noninvasive brain imaging techniques such as positron emission tomography and functional magnetic resonance imaging (fMRI) has accelerated the progress of connecting the effects of acupuncture with the central nervous system [1, 2]. Findings have revealed relationships between therapeutic acupuncture and brain activity in areas such as visual cortex [3, 4], language regions [5], limbic system, pain regions [1, 6–10], and somatosensory cortex [11]. While these results have shed some light on the issue of neuronal acupuncture specificity, defining an appropriate control when evaluating specificity is still controversial [12–16]. A sham point design that entails needling at the same depth and with the same pattern but at a nonacupoint located 10 millimeters away from the real one. However, Cho et al. reported that acupuncture is effective regardless of the choice of point at least for pain and analgesic response, which directly questioned the existence of point specificity [17]. Some studies also support the statement that differences between effects produced by sham and real acupuncture have remained unclear [18, 19].

The Siguan acupoint is a combination of bilateral LI4 (Hegu) and Liv3 (Taichong). In TCM, multiacupoint acupuncture is widely used to enhance the therapeutic effects as well as to avoid side effects. Siguan has been conventionally used for several symptoms, especially those of gastrointestinal and neurological disorders [20, 21]. Here, we chose to evaluate the multiacupoint specificity of Siguan using fMRI, which has rarely been reported. Based on results from studies using a single acupoint, we hypothesized that Siguan acupuncture may activate more specific brain regions than its sham point. We also expected to find both differences and commonalities between responses elicited by Siguan and its single acupoint components (Liv3/LI4). Thus, this study aimed to demonstrate whether multiacupoint acupuncture elicits specific activity in the human brain.

## 2. Materials and Methods

### 2.1. Subjects

Thirty-one healthy volunteers (14 men, 17 women; age range: 45–75 years; mean: 61.7 ± 7.9 years; all right-handed) were recruited after giving written informed consent with a basic understanding of this study. The inclusion criteria were as follows: (a) no neurological or psychiatric disorders such as stroke, depression, or epilepsy; (b) no neurological deficiencies such as visual or hearing loss; (c) no cognitive complaints; (d) no contraindications for MRI; and (e) no abnormal findings such as infarctions or focal lesions from conventional brain MRI. All the participants met the following exclusion criteria: (a) incapable of enduring pain or other physiology reaction caused by acupuncture stimulation and (b) severe head motion during scanning.

### 2.2. Stimuli and Scanning Procedure

MRI data acquisition was performed on a 3-Tesla scanner (Verio; Siemens, Erlangen, Germany). During scanning, hands and feet were exposed, and subjects were instructed to stay awake, hold still, keep eyes closed, and think nothing in particular. Functional images were acquired axially using an echo-planar imaging (EPI) sequence (repetition time [TR], 2000 ms, echo time [TE], 40 ms; flip angle [FA], 90°; field of view, 24 cm; image matrix, 64 × 64; slice number, 33; thickness, 3 mm; gap, 1 mm; bandwidth, 2232 Hz/pixel). Structural images, three-dimensional T1-weighted magnetization-prepared rapid gradient echo sagittal images, were also obtained (TR, 1900 ms; TE, 2.2 ms; inversion time [TI], 900 ms; FA, 90°; image matrix, 256 × 256; slice number, 176; thickness, 1 mm).

**Figure 1:**
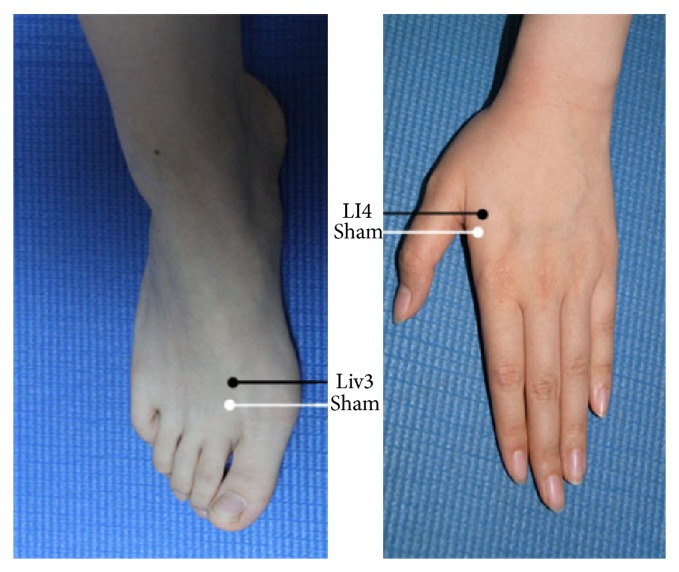
Anatomical location of Siguan and nearby sham acupoints: Liv3 (Taichong), LI4 (Hegu), and two sham points located 10 mm anterior to the corresponding real ones.

Subjects were randomly divided into real acupoint (21 subjects) and sham acupoint (10 subjects) groups. The real Siguan consists of four acupoints as mentioned above: Taichong (Liv3) on the dorsum of the left and right feet, in the depression anterior to the junction of the first and second metatarsals and Hegu (LI4) on the dorsum of the left and right hands, at the midpoint on the radial side of the second metacarpal. The sham acupoints were located approximately 10 mm to Liv3 or LI4 (Figure 1). We used stainless nonmagnetic needles that were 0.3 mm in diameter and 25 mm long. All acupuncture manipulations were performed by the same two skilled acupuncturists in synchrony. This ensured the consistency and accuracy of inserting needles into the four stimulation points at the same time during the scanning.

We adopted a 16-min scan time for the functional sequence. Here, our study used a single block experimental design to avoid the influence of unpredictable posteffects caused by acupuncture stimulation that may last several minutes to several hours. Baseline resting-state data were acquired in the initial 3 min. Then, fMRI scanning began while acupuncture stimulation was administered for 3 min. Needles were inserted (to the depth of 2 cm) into the four points simultaneously, with the needles rotated continuously (±180°, 60 times per minute) for both real and sham stimulation. Finally, needles were withdrawn and the scan continued acquiring data for 10 minutes.

### 2.3. Data Analysis

The preprocessing and data analysis were performed using analysis of functional neuroimages (AFNI) software (http://afni.nimh.nih.gov/). The first four images of each functional session were excluded from data processing to ensure image stabilization. The functional datasets of the two groups were preprocessed (corrected for slice acquisition time, corrected for motion, and spatially smoothed with a Gaussian filter of 6-mm full-width at half maximum). The realigned volumes were spatially standardized into the Talairach stereotaxic space by normalizing to the standard EPI template via their corresponding mean images. Then, all the normalized images were resliced into 3 × 3 × 3 mm voxels.

For the first level statistical analysis, we used the 3d Deconvolve program, part of the AFNI package, to analyze the impulse response functions on a voxelwise basis by modeling the response as a summation of tent-basis functions lasting 20 TRs (60 s) after acupuncture stimulation. The subject-specific contrast images were then used to perform the second level random-effects analysis. Then, two-sample *t*-tests were performed between data from real and sham acupoint groups. Multiple comparisons were analyzed using clustering and *P* value criteria established by AlphaSim software (http://afni.nimh.nih.gov/). Minimum cluster sizes were 17 voxels (459 mm^3^), and the maximum *P* value for voxels was 0.01. All surviving voxels had an adjusted *P* value < 0.05.

## 3. Results

### 3.1. Results of Acupuncture Stimulation at the Siguan Acupoint

**Figure 2:**
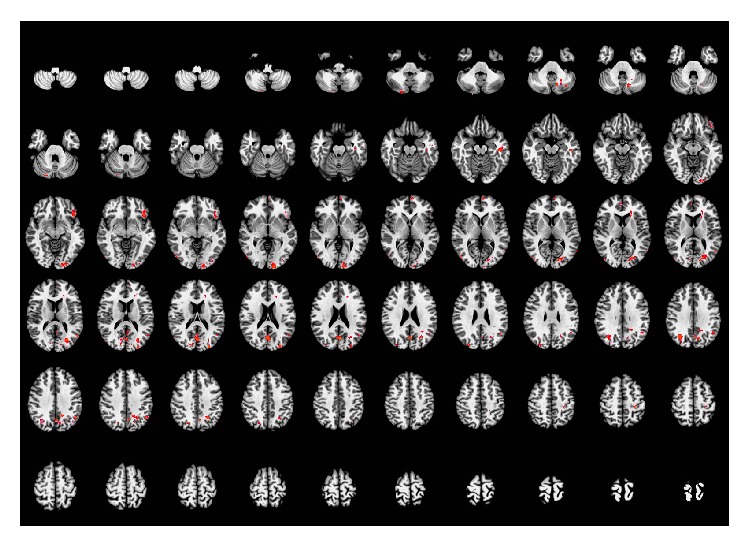
Brain regions activated by acupuncture stimulation at real acupoints. Left side of the images is the right side of the brain. *P* < 0.05 (AlphaSim correction).

Compared with the resting-state, acupuncture at the real acupoints activated brain regions primarily in the left calcarine gyrus, bilateral middle occipital gyrus (BA19), bilateral middle temporal gyrus (left BA 22), left inferior temporal gyrus, left inferior frontal gyrus, left superior medial prefrontal gyrus (left BA 10), right anterior cingulate cortex (right BA 10), left postcentral gyrus, left caudate nucleus, and the bilateral cerebellum (Crus 2 and VIII). This result is based on the anatomical location of the peak voxel in the activated cluster. When taking whole clusters into consideration, we also found higher activity in the right superior medial prefrontal gyrus (right BA 10), right calcarine gyrus, and the bilateral precuneus. The details of these regions are presented in Table 1 and Figure 2.

### 3.2. Results of Acupuncture Stimulation at the Sham Acupoint

**Figure 3:**
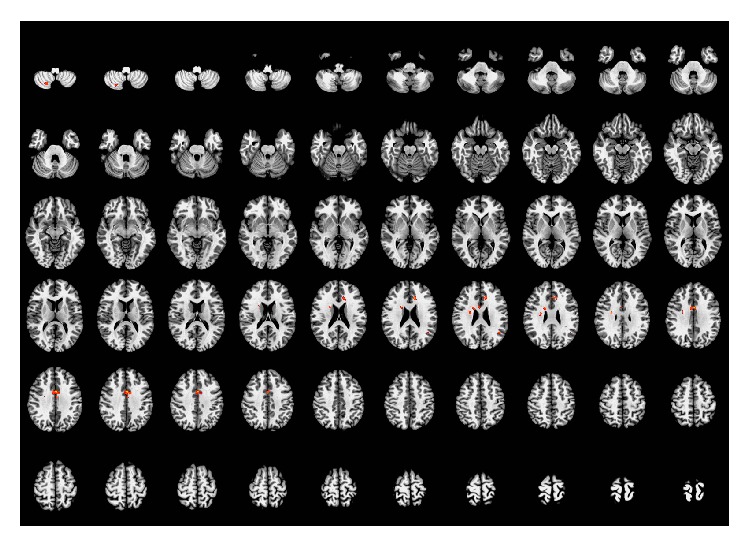
Brain regions activated by acupuncture stimulation at the sham point. Left side of the images is the right side of the brain. *P* < 0.05 (AlphaSim correction).

Compared with the resting-state, acupuncture at the sham acupoints activated brain regions primarily in the left anterior and middle cingulate cortices, right caudate nucleus, right insula, left angular gyrus, and right cerebellum (VIII). Considering the entire clusters, increased activity was also seen in right anterior and middle cingulate cortices. The details of these regions are presented in Table 2 and Figure 3.

### 3.3. Comparison between Real and Sham Acupuncture

**Figure 4:**
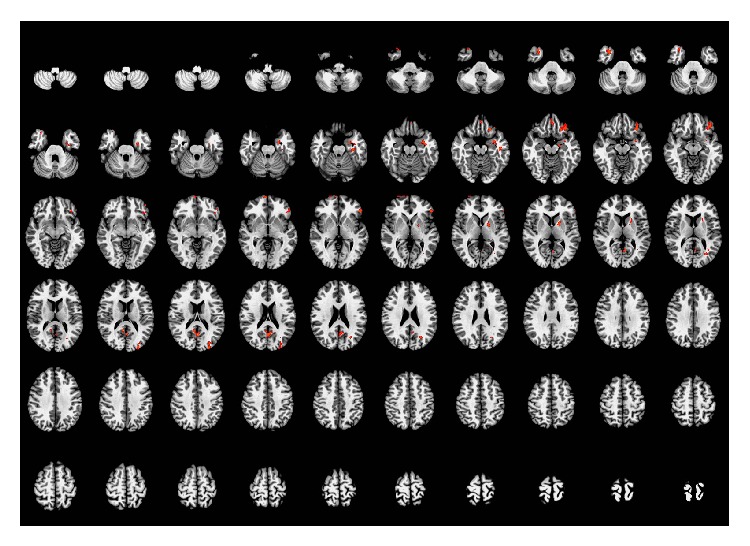
Brain regions activated more by acupuncture at the real acupoint than by acupuncture at the sham acupoint. Left side of the images is the right side of the brain. *P* < 0.05 (AlphaSim correction).

Brain regions that were activated more by real acupuncture stimulation than by sham point acupuncture were mostly located in the left inferior frontal gyrus, right superior medial prefrontal gyrus (right BA 10), left mid-orbital gyrus, right medial temporal pole (right BA 38), left parahippocampal gyrus, left precuneus (left BA 23), left fusiform gyrus (left BA 20), left pallidum, and left middle occipital gyrus. When taking whole voxels into account, increased signal was also present in the right precuneus (right BA 23), right fusiform gyrus, left putamen, right mid-orbital gyrus, bilateral rectal gyrus (BA 11), and the left inferior temporal gyrus. The details of these regions are presented in Table 3 and Figure 4.

## 4. Discussion

In general, the fMRI result shows increased brain activity after stimulation at Siguan and significant differences between real and sham point acupuncture. Many brain regions were activated, including somatosensory cortex (such as the superior parietal lobule and postcentral gyrus), limbic-paralimbic system (such as the calcarine gyrus, precuneus, cingulate cortex, and parahippocampal gyrus), visual-related cortex (such as the fusiform and occipital gyri), pain regions (such as basal ganglia), and the cerebellum. Some of these brain responses correlate with specific function areas. The middle occipital gyrus (BA 19) is visual cortex that has been reported to be activated by eye-related acupoints that affect vision, including Liv3 [3, 22]. The medial frontal prefrontal gyrus (BA10), superior parietal lobe, and the limbic system are regarded as visceral-related cerebral regions. In addition to these areas, other regions including the postcentral gyrus, a well-known somatosensory area [10, 13, 22–25], basal ganglia, and the cerebellum [10, 12–15] were generally activated upon stimulation, regardless of location. Yoo et al. suspected that activation of cerebellar loci could be explained by pain or possible hand motion caused by needling [14].

These findings support the idea that neuronal responses observed via fMRI are inclined to be unique and specific [19]. As Siguan is a combination of bilateral LI4 and Liv3, comparing brain activity during Siguan acupuncture to that during each single acupoint component should be informative. Indeed, studies on LI4 and Liv3 acupuncture have already identified activity in similar regions to those found here [8, 13, 22–25]. Liu et al. reported that stimulation of Liv3 selectively activated the middle occipital gyrus (BA 19), superior medial prefrontal gyrus (BA 10) [22]. Wu et al. found that Liv3 could elicit increased activity in the basal ganglia, which may have possibly been correlated with age [24]. Yan et al. reported that Liv3 acupuncture specifically activated more areas in the cerebellum, which is in accordance with its known effects on motor-related disorders [13]. Many studies have indicated that LI4 acupuncture induces activation in the insula, superior parietal lobule, middle temporal gyrus, and postcentral gyrus [13, 23]. Kong et al. found that the insula and putamen both were activated in two different types of simulation patterns at LI4 (electroacupuncture and manual acupuncture). They suggested that the insula activation occurs during the administration of pain while the putamen is essentially related to motor activity [23]. Yan et al. reported that LI4 and Liv3 acupuncture both activated bilateral middle temporal gyrus but that stimulation at LI4 could specifically elicit responses in the temporal pole [13].

The similarities suggest that brain activity induced by multiacupoint acupuncture may correlate closely with that of the individual acupoint components. In this way, single acupoint specificity can be extended further into multiacupoint specificity. Nevertheless, there are still some discrepancies between Siguan acupuncture and those of separate Liv3 and LI4 stimulation. The studies mentioned above showed that the thalamus and insula were closely associated with pain-related acupoints [12, 22, 25]. Fang et al. found increased thalamus activation at Liv3 when rotating the needle during acupuncture [12]. Claunch et al. reported activation of the anterior insula during LI4 stimulation was specific because the insula has roles in both visceral sensations and emotions [25]. However, in our present work, specific single acupoint responses in the insula and thalamus were not obviously seen after Siguan acupuncture, which indicates that brain activity after Siguan stimulation may not be a simple combination of activity resulting from Liv3 and LI4 stimulation.

Here, the sham point was also a combination of four separately located points. This allows a meaningful discussion of the activation results. The areas activated here by sham point stimulation, such as bilateral cingulate cortex, basal ganglia, insula, and cerebellum, are exactly part of the reported pain-related regions that are not thought to be related to any specific needling location. In this way, the sham multipoint did not show significant specificity. Therefore, the design and implementation of sham point need further investigation [18, 19].

One limitation of this study is that we did not focus on any reduction of brain activity that may have resulted from either real or sham stimulation. Actually, acupuncture at Liv3 and LI4 is reported to reduce activity in the limbic system and other pain-related brain areas, which is directly linked to their clinical use in analgesia [8, 13, 23, 25]. Although we paid more attention to increased activation, future work can attach more importance to deactivation specificity of multiacupoint acupuncture. Another limitation is that we did not perform any single acupoint stimulation design as an experimental group. In our present result, it seems that multiacupoint acupuncture may elicit specific activity beyond that of its individual components. Therefore, we expect that further studies can be performed to demonstrate this hypothesis.

## 5. Conclusion

Here, we focused on confirming the specificity of multiacupoint acupuncture using Siguan and a sham acupoint design. Extensive bilateral cortical and subcortical structures showed specific activation during Siguan stimulation while sham point only activated brain regions that are not thought to be related to specific needling location. Our findings suggest Siguan may elicit more specific and extensive activities in human brain than its sham point.

## Figures and Tables

**Table 1:** Brain regions activated by acupuncture stimulation at the real acupoint (Siguan) (compared with the resting-state).

Brain areas	BA	Side	Cluster size	Talairach			*t*-value
				*x*	*y*	*z*	
Calcarine gyrus		L	419	−1.5	−67.5	20.5	5.54
Middle occipital gyrus^*^		R	85	34.5	−61.5	29.5	5.39
Middle occipital gyrus	19	L	21	−28.5	−82.5	20.5	4.53
Middle temporal gyrus		R	42	46.5	−70.5	5.5	4.55
	22	L	79	−49.5	−58.5	14.5	4.29
Inferior temporal gyrus		L	39	−43.5	−19.5	−15.5	4.19
Inferior frontal gyrus		L	74	−40.5	22.5	−6.5	4.77
Superior medial prefrontal gyrus	10	L	29	−4.5	58.5	2.5	4.26
Anterior cingulate cortex	10	R	17	4.5	49.5	8.5	3.35
Postcentral gyrus		L	21	−28.5	−31.5	47.5	4.06
Caudate nucleus		L	37	−19.5	28.5	11.5	3.88
Cerebellum (crus 2)		L	41	−31.5	−67.5	−33.5	4.34
		R	28	22.5	−79.5	−27.5	4.47
Cerebellum (VIII)^*^		R	30	7.5	−49.5	−54.5	3.66

The peak voxel for each cluster and the corresponding name of the anatomical region are given. Asterisks indicate peak voxels located in the white matter. In these cases, we labeled the brain region in which most voxels in the cluster were located.

BA: Brodmann area. *P* < 0.05 (AlphaSim correction).

**Table 2:** Brain regions activated by acupuncture stimulation at the sham acupoint (compared with the resting-state).

Brain areas	Side	Cluster size	Talairach			*t*-value
			*x*	*y*		
Middle cingulate cortex	L	68	−1.5	4.5	32.5	8.17
Anterior cingulate cortex	L	28	−1.5	25.5	26.5	6.23
Insula^*^	R	19	25.5	−10.5	26.5	6.84
Angular gyrus	L	17	−40.5	−52.5	23.5	4.82
Caudate nucleus	R	28	22.5	4.5	20.5	4.56
Cerebellum (VIII)^*^	R	31	22.5	−61.5	−48.5	5.36

The peak voxel for each cluster and the corresponding name of the anatomical region are given. Asterisks indicate peak voxels located in the white matter. In these cases, we labeled the brain region in which most voxels in the cluster were located. *P* < 0.05 (AlphaSim correction).

**Table 3:** Brain regions activated more by stimulation at the real acupoint than by stimulation at the sham acupoint.

Brain areas	BA	Side	Cluster size	Talairach			*t*-value
				*x*	*y*	*z*	
Inferior frontal gyrus		L	116	−31.5	28.5	−12.5	3.30
Superior medial prefrontal gyrus	10	R	32	4.5	61.5	−0.5	3.18
Mid orbital gyrus		L	27	−1.5	37.5	−12.5	2.68
Medial temporal pole	38	R	39	31.5	13.5	−33.5	3.14
Parahippocampal gyrus		L	26	−28.5	−22.5	−18.5	3.12
Precuneus	23	L	68	−1.5	−58.5	17.5	3.53
Fusiform gyrus	20	L	76	−31.5	−10.5	−27.5	3.08
Pallidum		L	33	−19.5	−1.5	5.5	3.05
Middle occipital gyrus		L	80	−25.5	−88.5	17.5	3.17

The peak voxel for each cluster and the corresponding name of the anatomical region are given. In these cases, we labeled the brain region in which most voxels in the cluster were located.

BA: Brodmann area. *P* < 0.05 (AlphaSim correction).

## References

[1] Wu M. T., Hsieh J. C., Xiong J. (1999). Central nervous pathway for acupuncture stimulation: localization of processing with functional MR imaging of the brainpreliminary experience. *Radiology*.

[2] Cho Z. H., Oleson T. D., Alimi D., Niemtzow R. C. (2002). Acupuncture: the search for biologic evidence with functional magnetic resonance imaging and positron emission tomography techniques. *Journal of Alternative and Complementary Medicine*.

[3] Siedentopf C. M., Golaszewski S. M., Mottaghy F. M., Ruff C. C., Felber S., Schlager A. (2002). Functional magnetic resonance imaging detects activation of the visual association cortex during laser acupuncture of the foot in humans. *Neuroscience Letters*.

[4] Li G., Cheung R. T. F., Ma Q. Y., Yang E. S. (2003). Visual cortical activations on fMRI upon stimulation of the vision-implicated acupoints. *NeuroReport*.

[5] Li G., Liu H., Cheung R. T. F., Hung Y., Wong K. K. K., Shen G. G. X., Ma Q., Yang E. S. (2003). An fMRI study comparing brain activation between word generation and electrical stimulation of language-implicated acupoints. *Human Brain Mapping*.

[6] Hsieh J., Tu C., Chen F., Chen M., Yeh T., Cheng H., Wu Y., Liu R., Ho L. (2001). Activation of the hypothalamus characterizes the acupuncture stimulation at the analgesic point in human: a positron emission tomography study. *Neuroscience Letters*.

[7] Biella G., Sotgiu M. L., Pellegata G., Paulesu E., Castiglioni I., Fazio F. (2001). Acupuncture produces central activations in pain regions. *NeuroImage*.

[8] Hui K. K., Liu J., Makris N. (2000). Acupuncture modulates the limbic system and subcortical gray structures of the human brain: evidence from fMRI studies in normal subjects. *Human Brain Mapping*.

[9] Hui K. K. S., Liu J., Marina O., Napadow V., Haselgrove C., Kwong K. K., Kennedy D. N., Makris N. (2005). The integrated response of the human cerebro-cerebellar and limbic systems to acupuncture stimulation at ST 36 as evidenced by fMRI. *NeuroImage*.

[10] Wu M., Sheen J., Chuang K., Yang P., Chin S., Tsai C., Chen C., Liao J., Lai P., Chu K., Pan H., Yang C. (2002). Neuronal specificity of acupuncture response: a fMRI study with electroacupuncture. *NeuroImage*.

[11] Jeun S. S., Kim J. S., Kim B. S., Park S., Lim E., Choi G., Choe B. (2005). Acupuncture stimulation for motor cortex activities: a 3T fMRI Study. *The American Journal of Chinese Medicine*.

[12] Fang J. L., Krings T., Weidemann J., Meister I. G., Thron A. (2004). Functional MRI in healthy subjects during acupuncture: different effects of needle rotation in real and false acupoints. *Neuroradiology*.

[13] Yan B., Li K., Xu J., Wang W., Li K., Liu H., Shan B., Tang X. (2005). Acupoint-specific fMRI patterns in human brain. *Neuroscience Letters*.

[14] Yoo S., Teh E., Blinder R. A., Jolesz F. A. (2004). Modulation of cerebellar activities by acupuncture stimulation: evidence from fMRI study. *NeuroImage*.

[15] Zhang W. T., Jin Z., Luo F., Zhang L., Zeng Y., Han J. (2004). Evidence from brain imaging with fMRI supporting functional specificity of acupoints in humans. *Neuroscience Letters*.

[16] Na B., Jahng G., Park S., Jung W., Moon S., Park J., Bae H. (2009). An fMRI study of neuronal specificity of an acupoint: electroacupuncture stimulation of Yanglingquan (GB34) and its sham point. *Neuroscience Letters*.

[17] Cho Z. H., Hwang S. C., Wong E. K., Son Y. D., Kang C. K., Park T. S., Bai S. J., Kim Y. B., Lee Y. B., Sung K. K., Lee B. H., Shepp L. A., Min K. T. (2006). Neural substrates, experimental evidences and functional hypothesis of acupuncture mechanisms. *Acta Neurologica Scandinavica*.

[18] Campbell A. (2006). Point specificity of acupuncture in the light of recent clinical and imaging studies. *Acupuncture in Medicine*.

[19] Choi E. M., Jiang F., Longhurst J. C. (2012). Point specificity in acupuncture. *Chinese Medicine*.

[20] Yim Y. K., Kang W. C., Cho J. H., Shin J. W., Lee N. H., Choi S. M., Koo S. T., Park K. S., Son C. (2007). Crossover clinical trial to determine the effect of manual acupuncture at siguan points (Bilateral LI4 and LR3) on intestinal motility in healthy subjects. *The American Journal of Chinese Medicine*.

[21] Shin K., Park J., Lee S., Choi S., Ahn Y., Lee J., Kim J., Son C. (2013). Effect of siguan acupuncture on gastrointestinal motility: a randomized, sham-controlled, crossover trial. *Evidence-Based Complementary and Alternative Medicine*.

[22] Liu H., Xu J., Li L., Shan B., Nie B., Xue J. (2013). fMRI evidence of acupoints specificity in two adjacent acupoints. *Evidence-Based Complementary and Alternative Medicine*.

[23] Kong J., Ma L., Gollub R. L. (2002). A pilot study of functional magnetic resonance imaging of the brain during manual and electroacupuncture stimulation of acupuncture point (LI-4 Hegu) in normal subjects reveals differential brain activation between methods. *Journal of Alternative and Complementary Medicine*.

[24] Wu Y., Jin Z., Li K., Lu Z., Wong V., Han T., Zheng H., Caspi O., Liu G., Zeng Y., Zou L. (2010). Functional magnetic resonance imaging activation of the brain in children: real acupoint versus sham acupoint. *Journal of Child Neurology*.

[25] Claunch J. D., Chan S., Nixon E. E., Qiu W. Q., Sporko T., Dunn J. P., Kwong K. K., Hui K. K. S. (2012). Commonality and specificity of acupuncture action at three acupoints as evidenced by fMRI. *American Journal of Chinese Medicine*.

